# Freeze-Dried *Poecilobdella manillensis* Powder Regulates Cholesterol Homeostasis to Alleviate Hyperlipidemia

**DOI:** 10.3390/biom16081168

**Published:** 2026-08-11

**Authors:** Dezhi Yang, Qingmei Hu, Feng Shi, Xueling Chen, Yiquan Lin, Cuihua Fu, Fang Zhao, Xiaoju Zou, Xiaoxu Bi, Zichao Liu

**Affiliations:** 1 Engineering Research Center for Exploitation and Utilization of Leech Resources in Universities of Yunnan Province, School of Agriculture and Life Sciences, Kunming University, Kunming 650214, China; 2 School of Life Science and Engineering, Southwest Jiaotong University, Chengdu 610031, China; 3 Qujing Lichi Agricultural Technology Development Co., Ltd., Qujing 655100, China; 4 Yunnan International Joint Laboratory of Digital Conservation and Germplasm Innovation and Application of China-Laos Tea Resources, Pu’er University, Pu’er 665000, China; 5 College of Traditional Chinese Medicine, Yunnan University of Traditional Chinese Medicine, Kunming 650500, China; xiaojuzou@163.com

**Keywords:** hyperlipidemia, *Poecilobdella manillensis*, cholesterol homeostasis, CYP7A1, HMGCR, metabolomics

## Abstract

Hyperlipidemia (HL) is a major metabolic disorder and a critical risk factor for cardiovascular diseases, closely associated with oxidative stress, inflammation, and disrupted cholesterol homeostasis. Freeze-dried *Poecilobdella manillensis* powder (FDPMP), a traditional medicinal product, has shown therapeutic potential against hyperlipidemia; however, its underlying mechanisms remain largely unclear. In this study, HL was induced in ApoE^−/−^ mice by feeding a high-fat diet (HFD) for eight weeks, during which FDPMP or simvastatin (positive control) was orally administered daily. Concurrently, an in vitro foam cell model was established by exposing RAW264.7 macrophages to oxidized low-density lipoprotein (ox-LDL, 80 μg/mL) for 24 h, with FDPMP pretreatment applied 30 min prior to ox-LDL stimulation. Following intervention, serum lipid profiles, hepatic oxidative stress markers, histopathological changes, and cholesterol metabolism-related gene and protein expression were systematically evaluated. FDPMP administration significantly improved serum lipid profiles by reducing triglycerides, total cholesterol, and low-density lipoprotein cholesterol, while increasing high-density lipoprotein cholesterol levels. Additionally, FDPMP alleviated histopathological damage in the liver, kidney, and heart, enhanced antioxidant enzyme activities, and attenuated oxidative stress. Untargeted metabolomic analysis revealed that FDPMP markedly modulated key metabolic pathways, including choline metabolism, glycerophospholipid metabolism, and arachidonic acid metabolism. Mechanistically, FDPMP restored cholesterol homeostasis through dual regulation of cholesterol metabolism, characterized by upregulation of cholesterol 7α-hydroxylase (CYP7A1) to promote bile acid-mediated cholesterol excretion, alongside downregulation of 3-hydroxy-3-methylglutaryl-CoA reductase (HMGCR) and synthase (HMGCS1) to inhibit cholesterol biosynthesis. In vitro, FDPMP effectively suppressed ox-LDL-induced foam cell formation, reduced intracellular lipid accumulation, and mitigated oxidative stress in macrophages. Collectively, these findings demonstrate that FDPMP ameliorates hyperlipidemia through coordinated regulation of cholesterol synthesis and excretion, coupled with systemic metabolic reprogramming and antioxidative effects. This study provides mechanistic insights supporting FDPMP as a promising natural therapeutic candidate for hyperlipidemia and related metabolic disorders.

## 1. Introduction

Hyperlipidemia (HL) is a highly prevalent metabolic disorder characterized by elevated circulating lipids and represents a major modifiable risk factor for cardiovascular and cerebrovascular diseases, including atherosclerosis, myocardial infarction, and stroke [1]. Recent studies have increasingly recognized that hyperlipidemia itself drives atherosclerotic plaque pathology through multiple interconnected mechanisms: chronic lipid overload not only promotes cholesterol deposition within the vascular endothelium but also instigates oxidative stress, persistent vascular inflammation, and progressive endothelial dysfunction—a triad of pathological events that collectively initiate and accelerate the development of atherosclerotic lesions and systemic metabolic derangements [2,3,4,5]. Although the relationship between total dietary fat intake and cardiovascular risk remains incompletely understood, the deleterious effects of specific fat subtypes, particularly excessive consumption of saturated fats, have been consistently linked to disrupted lipid homeostasis and aggravated metabolic dysfunction [6], further supporting the view that HL pathogenesis involves a complex interplay of dietary, metabolic, and inflammatory factors.

At the cellular and molecular level, high-fat diet (HFD)-induced lipid overload promotes the oxidative modification of LDL. Under these pathological conditions, modified LDL (i.e., ox-LDL) redirects cholesterol transport toward excessive accumulation in macrophages, a process further exacerbated by dietary cholesterol oxidation products such as 7-ketocholesterol, which enhance macrophage cholesterol uptake and amplify pro-inflammatory signaling—together driving foam cell formation and vascular wall inflammation [7,8]. In parallel, emerging studies suggest that HFD-induced metabolic disorders are closely associated with gut microbiota dysbiosis and systemic metabolic reprogramming, which further exacerbate lipid dysregulation and inflammatory responses [9]. These findings collectively highlight that restoration of lipid metabolic homeostasis is a central therapeutic objective in HL management.

Cholesterol homeostasis is maintained through the coordinated regulation of biosynthesis, transport, and excretion pathways. Central to this regulatory network are 3-hydroxy-3-methylglutaryl-CoA reductase (HMGCR) and 3-hydroxy-3-methylglutaryl-CoA synthase 1 (HMGCS1), which function as key rate-limiting enzymes in the mevalonate pathway [10,11,12]. Their overactivation drives excessive endogenous cholesterol production, disrupting systemic lipid balance. In contrast, cholesterol 7α-hydroxylase (CYP7A1) catalyzes the rate-limiting step in the classical bile acid synthesis pathway, facilitating cholesterol catabolism and excretion [13]. Impaired CYP7A1 activity under HFD conditions suppresses bile acid synthesis and promotes cholesterol accumulation [14]. Given that elevated circulating cholesterol serves as the primary source for ox-LDL formation and subsequent foam cell development, a dual regulatory strategy that simultaneously inhibits cholesterol biosynthesis while enhancing bile acid-mediated elimination has emerged as an effective approach for restoring cholesterol homeostasis and alleviating HL.

*P. manillensis*, a traditional medicinal leech widely used in East Asian medicine, has long been applied in the treatment of cardiovascular and cerebrovascular disorders, including hypertension and thrombosis [15,16]. Its bioactive components, predominantly peptides and proteins, exhibit diverse pharmacological activities such as anticoagulant, thrombolytic, and analgesic effects [17,18]. Freeze-dried *P. manillensis* powder (FDPMP), which preserves these bioactive constituents, has been developed as a functional medicinal product and is increasingly utilized in clinical and health-related applications [19,20,21]. Previous studies have demonstrated that FDPMP exerts protective effects in metabolic disease models by inhibiting thrombin activity, suppressing inflammatory responses, and ameliorating tissue injury [20,21,22]. However, current mechanistic investigations have largely focused on its anticoagulant and anti-inflammatory properties, whereas whether FDPMP modulates lipid metabolism through the coordinated regulation of HMGCR/HMGCS1 and CYP7A1 remains unexplored. Exposure of macrophages to ox-LDL triggers their phenotypic transformation into lipid-laden foam cells, a hallmark event in early atherogenesis [23,24,25,26]. Accordingly, ox-LDL-stimulated RAW264.7 macrophages are widely employed as an in vitro model to investigate lipid uptake, oxidative stress, and foam cell formation in the context of HL and atherosclerosis [27].

In the present study, we comprehensively investigated the protective effects and underlying mechanisms of FDPMP against HFD-induced HL using ApoE^−/−^ mice and ox-LDL-stimulated RAW264.7 macrophages. By integrating biochemical assays, histopathological evaluation, untargeted metabolomics, and molecular analyses, we aimed to determine whether FDPMP ameliorates lipid metabolic dysfunction through coordinated regulation of cholesterol synthesis and excretion. Our results demonstrate that FDPMP restores cholesterol homeostasis by upregulating CYP7A1 while downregulating HMGCR and HMGCS1, thereby alleviating oxidative stress, inhibiting foam cell formation, and reducing tissue injury associated with HL. These findings provide mechanistic insights into the lipid-lowering effects of FDPMP and support its potential as a natural therapeutic candidate for HL and related metabolic disorders.

## 2. Materials and Methods

### 2.1. In Vivo Studies

#### 2.1.1. Preparation of Materials

FDPMP used in this study was provided by Yunnan Yuyao Biopharmaceutical Co., Ltd. (Yuyao, Qujing, China). and complied with the quality standards approved by the Yunnan Provincial Drug Administration.

Briefly, healthy *P. manillensis* specimens were maintained in clean water without feeding for 30 days to allow the elimination of residual gut contents and metabolic waste. During this period, water quality was strictly controlled under contaminant-free conditions, with temperature maintained at 24–26 °C, pH at 6.5–7.0, and dissolved oxygen at approximately 5 mg/L.

Following fasting, the specimens were pre-frozen in a freeze dryer (EYELA FDU-2100, Bunkyo City, Japan) at −80 °C for 1000 min, followed by sublimation drying at −80 °C and 5 Pa for 1000 min. Subsequently, desorption drying was performed at 40 °C and 5 Pa for 1000 min. The temperature ramp from sublimation drying to desorption drying was carried out over 1320 min under constant pressure of 5 Pa. The dried material was pulverized into a fine powder using a jet mill (Yangye Precision AM-100, Jiaxing, China) and passed through a vibrating sieve to remove coarse particles. The resulting powder was sterilized by ultraviolet irradiation for 30 min using a UV sterilizer (BIOBASE BJPX-SV200, Jinan; China) and packaged in sterile bottles, each containing 0.3 g of FDPMP.

The standard chow diet for mice contained ≤10% moisture, ≥18% crude protein, ≥4% crude fat, ≤5% crude fiber, ≤8% crude ash, 1.0–1.8% calcium, and 0.6–1.2% total phosphorus (Keao Xieli Feed Co., Ltd., Beijing, China). HFD consisted of 20% protein, 40% carbohydrates, and 40% fat (Beijing Keao Xieli Feed Co., Ltd., Beijing, China). Simvastatin was purchased from Shandong Lukang Pharmaceutical Group Setter Co., Ltd. (Taian, China).

#### 2.1.2. Experimental Animals and Grouping

Six-week-old female C57BL/6J mice and ApoE^−/−^ mice (license No. SCXK (Jing) 2024-0001) were obtained from Spefford Biotechnology Co., Ltd. (Beijing, China). All animals were housed in the Experimental Animal Center of Kunming University under controlled environmental conditions (temperature: 20–24 °C; relative humidity: 55–60%; 12 h light/dark cycle), with ad libitum access to food and water. The sample size was determined as six mice per group based on preliminary data and statistical power analysis. Using serum lipid parameters as the primary outcome variable, with an estimated effect size of 1.5 standard deviations, a significance level of α = 0.05, and a statistical power of 80%, the calculation indicated that 6–7 mice per group were required to detect biologically meaningful differences. This sample size is consistent with previous studies in genetically uniform ApoE^−/−^ mouse models under standardized experimental conditions, in which six mice per group have been widely adopted and proven sufficient to detect significant intergroup differences. The experimental protocol was reviewed and approved by the Experimental Animal Welfare Ethics Committee of Kunming University, and all procedures were conducted in strict accordance with the 3R principles (Replacement, Reduction, Refinement).

After a one-week acclimatization period, mice were randomly assigned into six groups (*n* = 6 per group): (1) Control group: C57BL/6J mice fed a standard diet and administered saline; (2) model group: ApoE^−/−^ mice fed a high-fat diet (HFD) and administered saline; (3) simvastatin group: ApoE^−/−^ mice fed an HFD and treated with simvastatin (10 mg/kg/day); (4) low-dose FDPMP group (LDFDPMP): ApoE^−/−^ mice fed an HFD and treated with FDPMP at 0.0043 g/kg/day (dissolved in sterile water); (5) medium-dose FDPMP group (MDFDPMP): ApoE^−/−^ mice fed an HFD and treated with FDPMP at 0.0086 g/kg/day (dissolved in sterile water); and (6) high-dose FDPMP group (HDFDPMP): ApoE^−/−^ mice fed an HFD and treated with FDPMP at 0.0172 g/kg/day (dissolved in sterile water). All treatments were administered by oral gavage once daily. The FDPMP doses were calculated based on the human clinical dose (0.3 g/60 kg) by body weight conversion, corresponding to 0.86-, 1.72-, and 3.44-fold of the human equivalent dose, respectively [28]. All FDPMP solutions were freshly prepared in sterile water before each administration.

#### 2.1.3. Sample Collection

After eight weeks of treatment, mice were fasted for 12 h and anesthetized by intraperitoneal injection of 2% sodium pentobarbital (50 mg/kg; Sigma, St. Louis, MO, USA). Blood samples were collected from the carotid artery and centrifuged at 3000× *g* rpm for 10 min at 4 °C to obtain plasma, which was stored at −80 °C for subsequent analyses. Following blood collection, mice were euthanized, and the liver, heart, and kidneys were rapidly excised and rinsed with cold saline. The liver was divided into portions for biochemical, histopathological, and molecular analyses, while the heart and kidneys were processed exclusively for histopathological evaluation. All tissues were stored at −80 °C until further use.

#### 2.1.4. Metabolomic Analysis: QC Procedures and Metabolite Identification

To ensure data quality, pooled quality control (QC) samples were prepared by mixing equal volumes of all plasma samples and injected regularly throughout the analytical run to monitor instrument performance and reproducibility. Metabolite features with a relative standard deviation (RSD) > 30% in QC samples were excluded from further analysis. Missing values were imputed using half of the minimum detected value for each compound. To reduce the false discovery rate in multiple comparisons, the Benjamini–Hochberg method was applied, and metabolites with an adjusted *p*-value (q-value) < 0.05 were considered statistically significant. Metabolite identification was performed by matching MS/MS spectra against an in-house database and public libraries (e.g., HMDB, METLIN). Following the Metabolomics Standards Initiative (MSI) guidelines, metabolites were classified as Level 2 (putatively annotated compounds), as authentic standards were not available for confirmation in this untargeted screening. Targeted validation of candidate metabolites using authentic standards is planned for future studies.

#### 2.1.5. Biochemical Analyses

Plasma levels of total cholesterol (TC), triglycerides (TG), high-density lipoprotein cholesterol (HDL-C), and low-density lipoprotein cholesterol (LDL-C) were measured using commercial assay kits (Sangon Biotech, Shanghai; China) according to the manufacturer’s instructions.

For hepatic biochemical analysis, liver tissues were homogenized in appropriate buffer and centrifuged to collect supernatants. The levels of malondialdehyde (MDA), glutathione peroxidase (GSH-Px), and superoxide dismutase (SOD) were determined using corresponding assay kits (Sangon Biotech, Shanghai, China). All measurements were performed in triplicate, and absorbance values were recorded using a microplate reader.

#### 2.1.6. Histological Analysis (H&E Staining)

Liver, kidney, and heart tissues were fixed in 4% paraformaldehyde for 48 h, followed by dehydration through a graded ethanol series (30–100%). The tissues were then cleared with xylene and embedded sequentially in soft paraffin (60 °C for 30 min) and hard paraffin (65 °C for 60 min). Paraffin-embedded tissues were sectioned at a thickness of 5 μm. The sections were deparaffinized and rehydrated, then stained with hematoxylin for nuclear visualization and eosin for cytoplasmic staining. After staining, the sections were dehydrated, cleared, and mounted. Tissue morphology was observed and images were captured using a light microscope. Specifically, liver sections were examined for hepatocellular necrosis, steatosis, and inflammatory cell infiltration; kidney sections were assessed for glomerular atrophy, tubular dilation, and interstitial inflammation; and heart sections were evaluated for myocardial fiber arrangement, interstitial edema, and inflammatory cell infiltration.

#### 2.1.7. Western Blot Analysis

Approximately 30 mg of liver tissue was ground in liquid nitrogen and lysed with 500 μL of lysis buffer supplemented with protease inhibitor (lysis buffer: protease inhibitor = 100:1). The lysates were centrifuged at 12,000× *g* rpm for 10 min at 4 °C, and the supernatants were carefully collected. Protein concentrations were determined using the bicinchoninic acid (BCA) assay.

Equal amounts of protein (20 μg per sample) were separated by 10% SDS–PAGE and transferred onto polyvinylidene difluoride (PVDF) membranes using a wet transfer system (270 mA for 60 min). Membranes were blocked with 5% skim milk in 1× TBST at room temperature for 1 h and then incubated overnight at 4 °C with primary antibodies against CYP7A1, HMGCR, and HMGCS1 (1:1000; Affinity, Changzhou, China). After washing five times with 1× TBST, the membranes were incubated with a rhodamine-labeled goat anti-rabbit IgG secondary antibody (1:5000; ZSGB-BIO, Beijing, China) for 1 h at room temperature.

Protein bands were visualized using an enhanced chemiluminescence (ECL) detection system and imaged with a chemiluminescence gel imaging system. Band intensities were quantified using Image software Fiji (National Institutes of Health, Bethesda, MD, USA), with GAPDH used as an internal loading control for semi-quantitative analysis.

#### 2.1.8. Quantitative Real-Time PCR

Total RNA was extracted from liver tissues using an RNA extraction kit (Omega, Connecticut, CT, USA) according to the manufacturer’s instructions. RNA concentration and purity were assessed using a NanoDrop 2000 spectrophotometer (Thermo Fisher Scientific, Waltham, MA, USA). Complementary DNA (cDNA) was synthesized using a reverse transcription kit (Takara, Beijing, China).

For quantitative real-time PCR analysis, 2 ng of cDNA was added to the SYBR Green-based qPCR reaction system, and the mRNA expression levels of CYP7A1, HMGCR, and HMGCS1 were determined using a SYBR Green qPCR kit (Roche, Basel, Switzerland) on a LightCycler^®^ 480 system (Roche Applied Science, Rotkreuz; Switzerland). GAPDH was used as the internal reference gene. The primer sequences used for amplification are listed in Table 1.

The qPCR reaction mixture consisted of 10 μL of 2× ChamQ SYBR Color qPCR Master Mix (Beyotime, Shanghai, China), 0.8 μL of each forward and reverse primer, 0.4 μL of 50× ROX Reference Dye (Solarbio, Beijing, China), 6 μL of ddH_2_O, and 2 μL of diluted cDNA. The thermal cycling conditions were as follows: initial denaturation at 95 °C for 5 min, followed by 40 cycles of denaturation at 95 °C for 30 s, annealing at 55 °C for 30 s, and extension at 72 °C for 40 s [29]. Relative gene expression levels were calculated using the comparative Ct method (2^−ΔΔCt^) [30].

#### 2.1.9. Immunofluorescence of Tissue Sections

Paraffin-embedded tissue sections were deparaffinized and rehydrated, followed by antigen retrieval using citrate buffer (pH 6.0–6.4) under high-pressure conditions (600 W, medium heat, 15 min), and then cooled to room temperature for 1 h. Non-specific binding was blocked with 5% bovine serum albumin (BSA) for 1 h at room temperature.

Sections were incubated overnight at 4 °C with primary antibodies against CYP7A1, HMGCR, and HMGCS1 (1:300; Affinity, Liyang, China) diluted in 5% BSA. After washing with PBST, the sections were incubated with fluorescently labeled secondary antibodies (1:500; ZSGB-BIO, Beijing, China) diluted in 5% BSA for 1–2 h at room temperature. Nuclei were counterstained with DAPI, and sections were mounted with an anti-fade mounting medium. Fluorescence images were acquired using a confocal laser scanning microscope.

### 2.2. In Vitro Studies

#### 2.2.1. Immunofluorescence

Cells were fixed with 4% paraformaldehyde for 20 min at room temperature, washed with PBS, and blocked with 5% BSA for 1 h. The cells were then incubated overnight at 4 °C with primary antibodies diluted in 5% BSA. After three washes with PBS, fluorescently labeled secondary antibodies were added and incubated for 1 h at room temperature. Following PBS washes, nuclei were stained with DAPI, and the cells were mounted with an anti-fade reagent and observed under a confocal microscope.

#### 2.2.2. Cell Culture and Treatment

RAW264.7 macrophages were cultured in Dulbecco’s modified Eagle’s medium (DMEM) supplemented with 10% fetal bovine serum and maintained at 37 °C in a humidified incubator containing 5% CO_2_. Cells were passaged the following day, and cells in the logarithmic growth phase were used for subsequent experiments.

The cells were divided into the following groups: blank control group; model group treated with oxidized low-density lipoprotein (ox-LDL, 80 μg/mL); simvastatin groups treated with simvastatin (2.5, 5, and 10 μM) in combination with ox-LDL (80 μg/mL); and FDPMP groups treated with FDPMP (5, 10, 20, 40, 80, 160, 320, and 640 mg/L) in combination with ox-LDL (80 μg/mL). Cells in the simvastatin and FDPMP groups were pretreated with the corresponding compounds for 30 min, followed by incubation with ox-LDL for 24 h. The blank control group received an equal volume of culture medium without any treatment.

#### 2.2.3. Cell Viability Assay (CCK-8)

RAW264.7 macrophages in optimal growth conditions (approximately 80–90% confluence) were harvested, centrifuged, and resuspended. The cell density was adjusted to 1 × 10^5^ cells/mL, and 200 μL of the cell suspension was seeded into 96-well plates. After incubation for 12 h, cells were treated with different concentrations of ox-LDL (Yiyuan Biotechnologies, Fuzhou, China), FDPMP, and simvastatin as indicated.

Following 24 h of treatment, 20 μL of CCK-8 reagent (Biosharp, Beijing, China) was added to each well, and the plates were incubated in the dark at 37 °C in a 5% CO_2_ incubator for 2 h. Absorbance was measured at 450 nm using a microplate reader. Cell viability was calculated based on the absorbance values at different treatment concentrations.

#### 2.2.4. Foam Cell Formation Analysis

Intracellular lipid accumulation and foam cell formation were evaluated using aggregation-induced emission (AIE) (EPRUI Biotech, Shanghai, China) fluorescence staining with lipid-specific yellow and blue probes.

The AIE-LD-Y01 yellow probe stock solution was stored in the dark at −20 °C. Prior to use, 1 μL of the stock solution was diluted in 1–2 mL of cell culture medium or PBS to prepare a 10 μM working solution. Cells were incubated with the working solution for 30 min at room temperature, followed by three washes with PBS. Fluorescence images were acquired using a confocal or fluorescence microscope with an excitation wavelength of 488 nm and emission collected between 550 and 650 nm.

The AIE-LD-B01 blue probe stock solution was also stored in the dark, and a 100 nM working solution was freshly prepared before use. Both live and fixed cells were incubated with the working solution for 30 min, followed by PBS washing. Fluorescence signals were observed using a confocal or fluorescence microscope with an excitation wavelength of 405 nm and emission collected between 418 and 500 nm. These procedures enabled visualization and qualitative assessment of intracellular lipid accumulation and foam cell formation.

### 2.3. Metabolomic Analysis

#### 2.3.1. Sample Preparation

For metabolomic analysis, 50 μL of plasma was mixed with 200 μL of extraction solvent (methanol/acetonitrile, 1:1, *v*/*v*) containing isotope-labeled internal standards. Samples were vortexed for 30 s, sonicated in an ice-water bath for 10 min, and incubated at −40 °C for 1 h to precipitate proteins.

After centrifugation at 12,000× *g* rpm for 15 min at 4 °C, the supernatants were collected and transferred to vials for analysis. Untargeted metabolomic profiling was performed by Shanghai Personalbio Biotechnology Co., Ltd. (Shanghai, China).

#### 2.3.2. HPLC-QTOF/MS Analysis

Metabolomic analysis was performed using an ACQUITY UPLC BEH AMIDE column (2.1 mm × 100 mm, 1.7 μm; Waters Corporation, Milford, MA, USA). The mobile phases consisted of solvent A (H_2_O containing formic acid) and solvent B (acetonitrile). The gradient elution program was as follows: 0–1 min, 95% A; 1–12 min, linear decrease from 95% to 5% A; 12–13.5 min, 5% A; 13.5–13.6 min, 5% to 95% A; and 13.6–16 min, 95% A. The flow rate was set at 300 μL/min, with an injection volume of 6 μL, and the autosampler temperature was maintained at 4 °C.

Mass spectrometric detection was performed in both positive and negative ion modes. In positive ion mode, the heater temperature was set to 300 °C, and the sheath, auxiliary, and sweep gas flow rates were 45, 15, and 1 arbitrary units, respectively. The electrospray ionization voltage was 3.0 kV, the capillary temperature was 350 °C, and the S-lens RF level was set to 30%. In negative ion mode, the operating conditions were similar, except that the electrospray voltage was set to 3.2 kV and the S-lens RF level was adjusted to 60%.

Data acquisition was conducted using full-scan mode (*m*/*z* 70–1050) combined with data-dependent tandem mass spectrometry (dd-MS^2^, top N = 10). The mass resolution was set to 70,000 for MS^1^ and 17,500 for MS^2^. High-energy collision-induced dissociation (HCD) was applied for fragmentation.

#### 2.3.3. Metabolomic Data Processing and Analysis

Raw mass spectrometry data were converted to mzXML format using ProteoWizard 2 software. Subsequent data processing, including retention time correction, peak detection, extraction, integration, and alignment, was performed using the XCMS package, with the minFrac parameter set to 0.5 and a cut-off value of 0.6. Metabolite identification was carried out using a custom R-based workflow in combination with a self-built secondary mass spectrometry database.

After data preprocessing, multivariate statistical analyses were conducted, including principal component analysis (PCA) and orthogonal partial least squares discriminant analysis (OPLS-DA). One-way analysis of variance (ANOVA) was performed to assess statistical significance, with *p* < 0.05 considered significant. Differential metabolites were screened based on OPLS-DA results using a variable importance in projection (VIP) threshold > 1.00. Identified differential metabolites were further mapped to metabolic pathways using the KEGG database and other relevant resources to explore their biological significance [31].

#### 2.3.4. Statistical Analysis

Statistical analyses were performed using SPSS version 27.0 (IBM, Armonk, NY, USA). Data normality was assessed using the Shapiro–Wilk test, and homogeneity of variances was evaluated using Levene’s test. For data meeting the assumptions of normality and homogeneity, differences among groups were analyzed using one-way analysis of variance (ANOVA), followed by Tukey’s post hoc test for multiple comparisons. A *p* value < 0.05 was considered statistically significant. All data were presented as mean ± standard deviation (SD). Graphs were generated using Origin 2021 software (OriginLab, Northampton, MA, USA).

## 3. Results

### 3.1. Effects of FDPMP on Body Weight, Lipid Profile, and Hepatic Oxidative Stress in HFD-Induced Hyperlipidemic Mice

**Figure 1:**
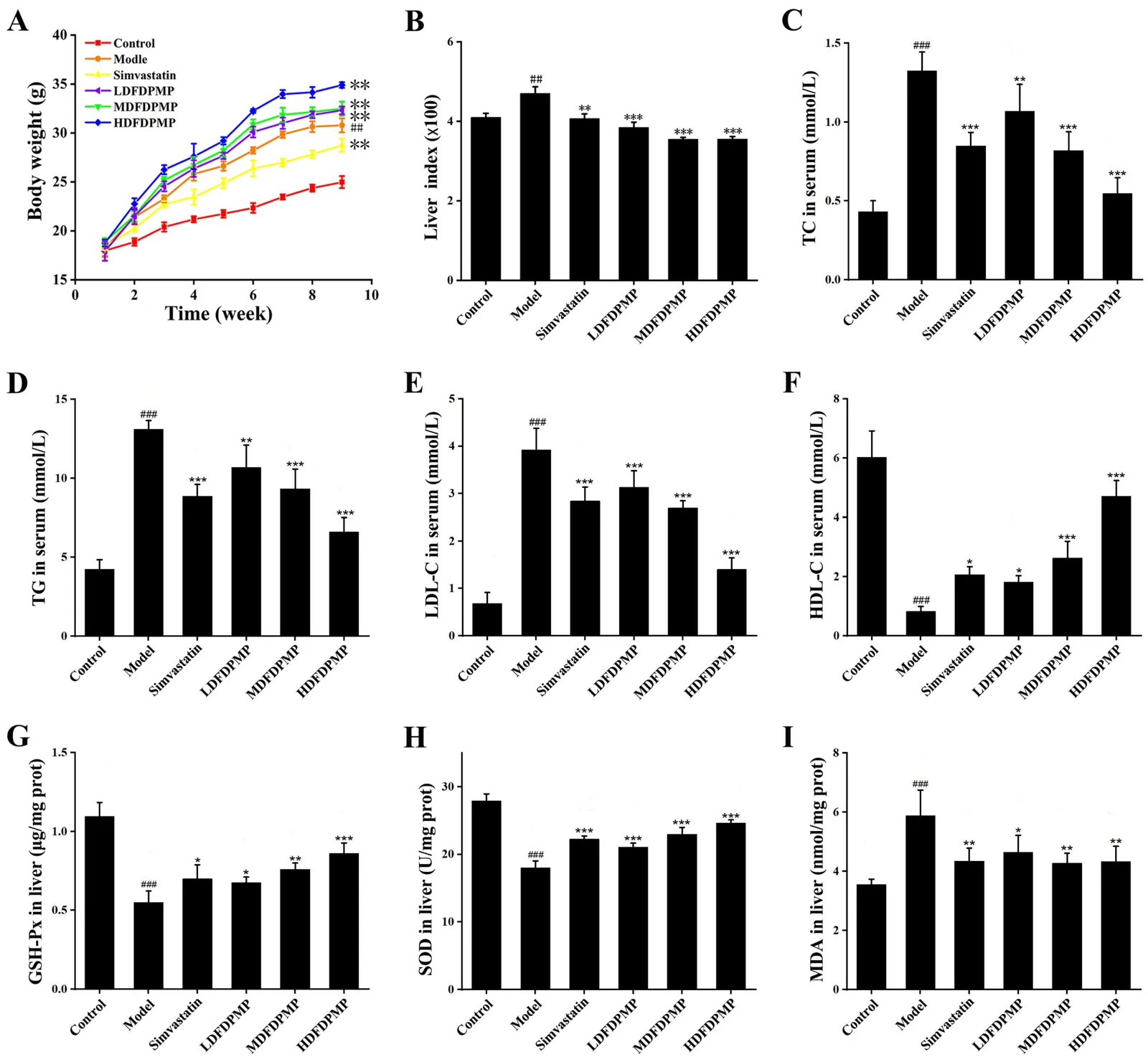
Effects of FDPMP on body weight, liver index, serum lipid levels, and hepatic oxidative stress in HFD-induced hyperlipidemic mice (*n* = 6). (**A**) Body weight; (**B**) Liver index; (**C**–**F**) Serum lipid parameters, including TG, TC, HDL-C, and LDL-C; (**G**–**I**) Hepatic oxidative stress markers, including GSH-Px, SOD, and MDA. Note: Compared with the control group: ^##^ *p* < 0.01, ^###^ *p* < 0.001. Compared with model group: * *p* < 0.05, ** *p* < 0.01, *** *p* < 0.001.

After eight weeks of treatment, mice in the model group exhibited a significant increase in body weight compared with the control group. In contrast, simvastatin treatment significantly reduced body weight, whereas FDPMP administration resulted in a dose-dependent increase in body weight (Figure 1A).

The liver index was markedly elevated in HFD-fed mice relative to the control group, indicating hepatic enlargement. Notably, FDPMP treatment, particularly at high doses, significantly reduced the liver index compared with the model group (Figure 1B).

Serum lipid analysis showed that HFD feeding significantly increased levels of triglycerides (TG), total cholesterol (TC), and low-density lipoprotein cholesterol (LDL-C), while decreasing high-density lipoprotein cholesterol (HDL-C). Both simvastatin and FDPMP treatments effectively improved these lipid abnormalities. Specifically, FDPMP significantly decreased TG, TC, and LDL-C levels and increased HDL-C levels in a dose-dependent manner, although values in the high-dose group remained significantly different from those in the control group (Figure 1C–F).

Assessment of hepatic oxidative stress markers revealed that HFD significantly decreased the activities of glutathione peroxidase (GSH-Px) and superoxide dismutase (SOD), while increasing malondialdehyde (MDA) levels compared with the control group. Treatment with simvastatin and FDPMP significantly reversed these changes. FDPMP exhibited a dose-dependent effect, progressively restoring GSH-Px and SOD activities and reducing MDA levels (Figure 1G–I). These results indicate that FDPMP alleviates lipid abnormalities and oxidative stress in HFD-induced hyperlipidemic mice.

### 3.2. Multivariate Statistical Analysis of Serum Metabolites

**Figure 2:**
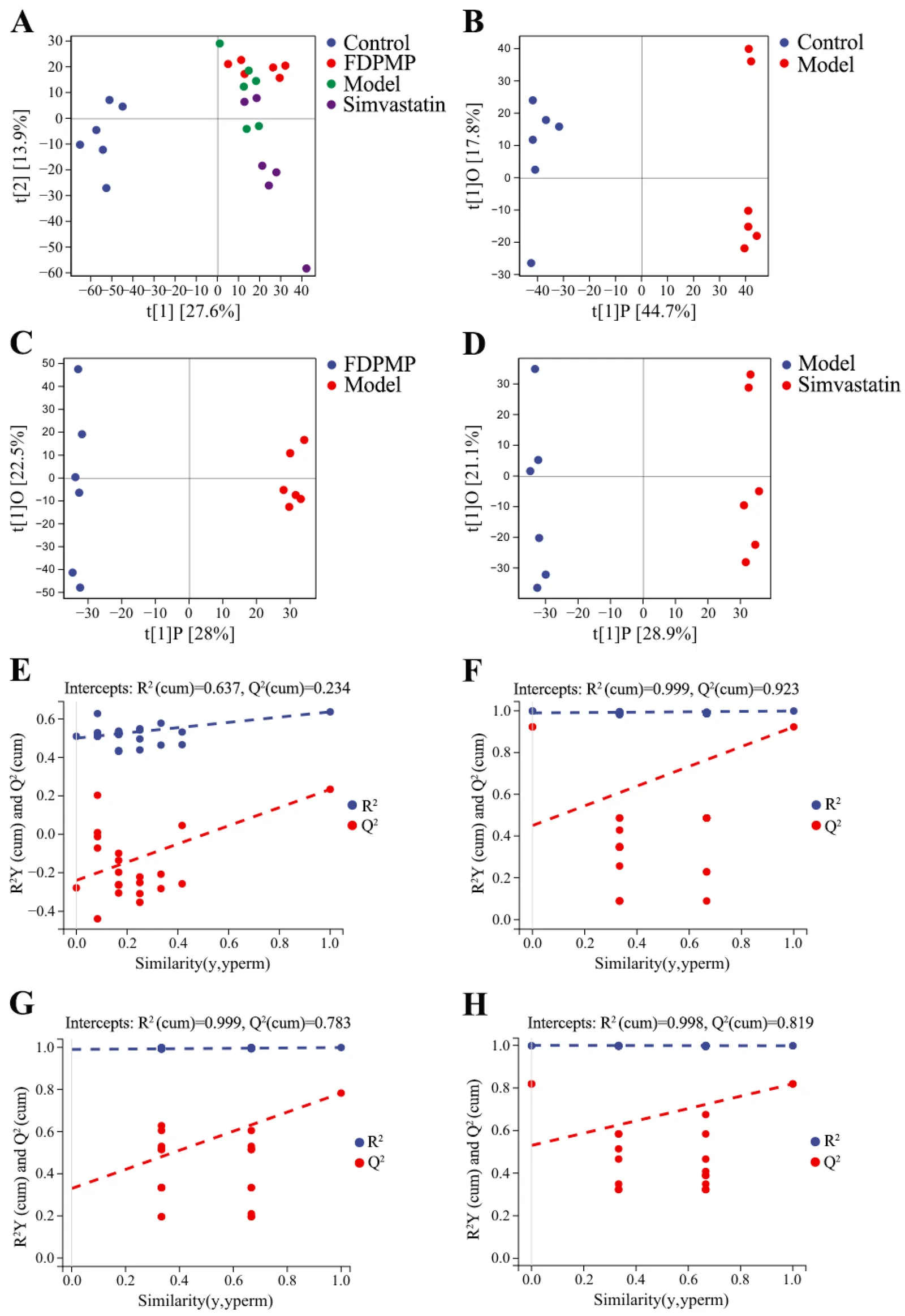
Multivariate statistical analysis of serum metabolomic profiles (*n* = 6). (**A**) Principal component analysis (PCA) score plot. (**B**–**D**) Orthogonal partial least squares discriminant analysis (OPLS-DA) score plots for pairwise group comparisons. (**E**–**H**) Permutation tests (200 iterations) validating OPLS-DA models.

Principal component analysis (PCA) demonstrated clear separation among the control, model, FDPMP, and simvastatin groups, indicating distinct metabolic profiles and substantial alterations induced by HFD (Figure 2A).

To further characterize metabolic differences, orthogonal partial least squares discriminant analysis (OPLS-DA) was performed. The score plots showed distinct separation between the control and model groups, as well as between the model group and the FDPMP- and simvastatin-treated groups, suggesting that both interventions partially reversed HFD-induced metabolic perturbations (Figure 2B–D).

The OPLS-DA models exhibited strong explanatory and predictive performance, with R^2^X, R^2^Y, and Q^2^ values indicating robust model reliability. Permutation tests (200 iterations) further confirmed the validity of the models, as evidenced by lower Q^2^ values in permuted models and negative or low intercepts, indicating no overfitting (Figure 2E–H). Those results demonstrate that FDPMP treatment significantly modulates the serum metabolomic profile altered by HFD.

The clustering and separation of groups indicate distinct metabolic profiles and the effects of FDPMP and simvastatin on HFD-induced metabolic alterations.

### 3.3. Effects of FDPMP on Metabolic Pathways in HFD-Induced Hyperlipidemic Mice

**Figure 3:**
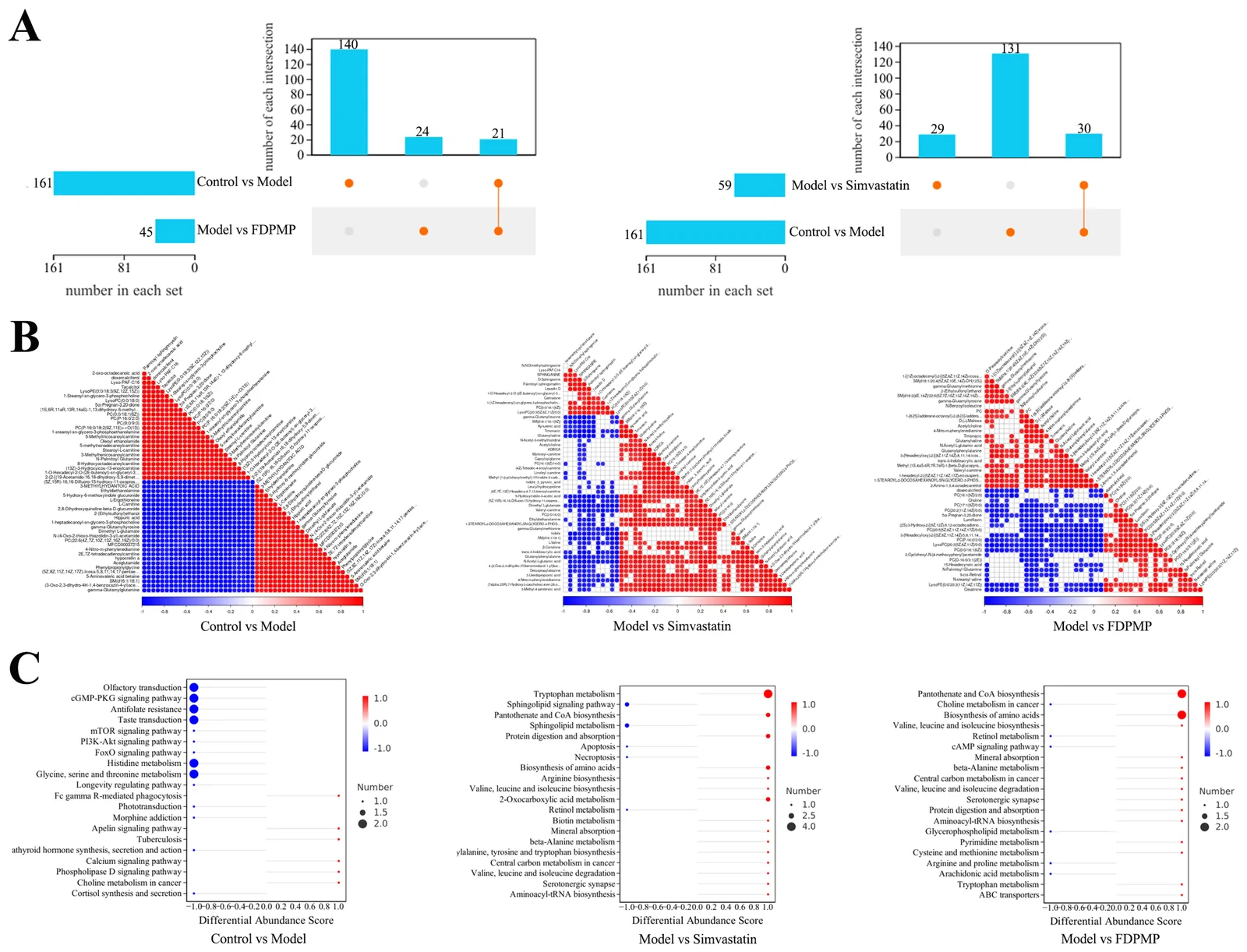
Identification of differential metabolites and metabolic pathway analysis (*n* = 6). (**A**) Heatmap of differential metabolites identified based on VIP > 1 and *p* < 0.05. (**B**) Correlation network analysis of metabolites; (**C**) KEGG pathway enrichment analysis.

Based on variable importance in projection (VIP > 1) and *p* < 0.05, a total of 21 differential metabolites were identified between the control, model, and FDPMP groups, whereas 30 differential metabolites were identified in comparisons involving the simvastatin group (Figure 3A).

The identified metabolites were classified into multiple categories, including lipids and lipid-like molecules, amino acids and derivatives, organic acids, carbohydrates, and organoheterocyclic compounds. Compared with the control group, HFD significantly altered metabolites associated with fatty acyls, lipids, and carboxylic acids. Following FDPMP treatment, glycerophosphocholines were markedly downregulated, suggesting reduced phospholipid turnover and inflammatory signaling. Lipids and lipid-like molecules were significantly decreased, consistent with the overall lipid-lowering effects of FDPMP. Fatty acid esters and amino acid-related metabolites were upregulated, which may reflect enhanced fatty acid metabolism and improved antioxidant capacity (Table 2). Of note, three features (Nos. 1, 2, and 18) could not be confidently annotated using available spectral libraries and were therefore excluded from further pathway interpretation. All remaining metabolites listed in Table 2 are reported as putatively annotated compounds (Level 2) according to the Metabolomics Standards Initiative (MSI) guidelines, as authentic standards were not used for confirmation in this untargeted screening. Importantly, while these metabolomic alterations support a FDPMP-associated shift in systemic lipid metabolism, they are correlative in nature and do not, on their own, constitute direct evidence of enhanced cholesterol excretion or reduced biosynthesis. Direct functional measurements—such as serum and hepatic bile acids, fecal bile acid output, fecal neutral sterols, and hepatic cholesterol content—are required to definitively establish these mechanistic conclusions. Nevertheless, the observed changes in glycerophosphocholines and lipid-related metabolites are consistent with and complement the molecular findings regarding CYP7A1 upregulation and HMGCR/HMGCS1 downregulation.

Correlation analysis revealed that HFD markedly disrupted metabolic associations, particularly enhancing positive correlations among lipid metabolites while altering the relationships between lipid and amino acid metabolites. These alterations were attenuated following FDPMP treatment, indicating partial restoration of metabolic coordination (Figure 3B).

KEGG pathway analysis further demonstrated that HFD significantly upregulated lipid-related pathways, including choline metabolism. FDPMP treatment markedly downregulated these pathways and significantly affected additional pathways, such as glycerophospholipid metabolism, arachidonic acid metabolism, retinol metabolism, cAMP signaling, and arginine and proline metabolism (Figure 3C).

Collectively, these results indicate that FDPMP modulates multiple metabolic pathways associated with lipid metabolism and systemic metabolic homeostasis.

### 3.4. Protective Effects of FDPMP on HFD-Induced Histopathological Alterations

Hematoxylin and eosin (H&E) staining revealed significant histopathological changes in multiple organs of HFD-fed mice, which were alleviated by FDPMP treatment to varying degrees, indicating its broad-spectrum tissue-protective effects.

**Figure 4:**
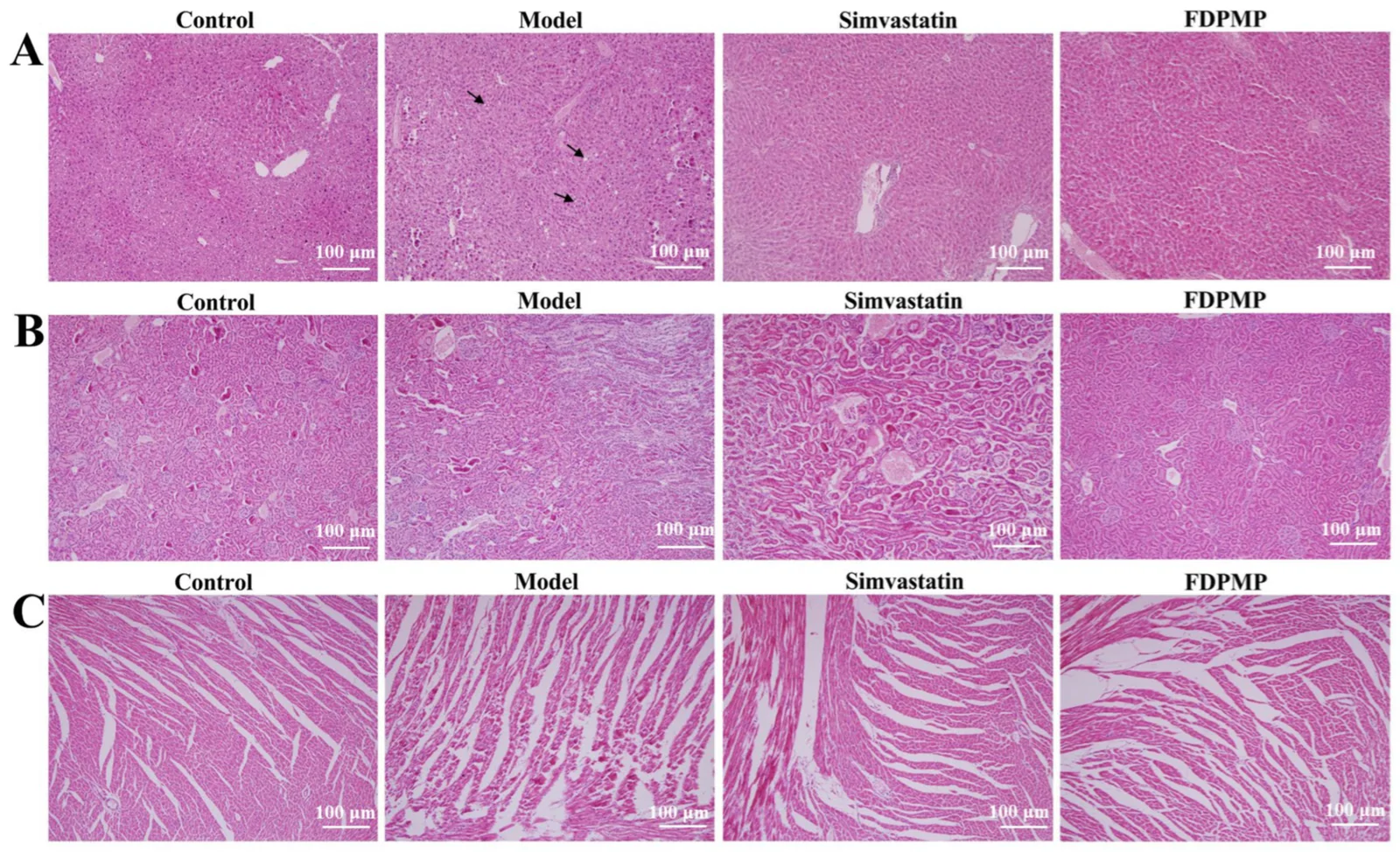
Histopathological analysis of liver, kidney, and heart tissues (*n* = 6). Representative hematoxylin and eosin (H&E) staining images of (**A**) liver, (**B**) kidney, and (**C**) heart tissues from different experimental groups. Note: Black arrows indicate hepatic steatosis, hepatocellular vacuolization, and disruption of the hepatic sinusoidal architecture.

In the liver, the model group exhibited pronounced steatosis, hepatocellular vacuolization, and disrupted sinusoidal architecture compared with the control group, suggesting that prolonged high-fat diet feeding induced severe hepatic lipid accumulation and parenchymal injury. These pathological changes were partially alleviated by simvastatin and markedly improved by FDPMP treatment, with hepatocyte morphology approaching normal, sinusoidal architecture restored, and liver morphology closely resembling that of the control group (Figure 4A), demonstrating that FDPMP exerts potent protective effects against HFD-induced liver injury.

In the kidney, HFD induced tubular edema, epithelial cell degeneration, protein deposition, and glomerular abnormalities, indicating that high-fat overload could trigger significant structural and functional damage to the kidney. Both simvastatin and FDPMP treatments mitigated these pathological features, with FDPMP showing more pronounced improvements in the restoration of tubular integrity and interstitial structure (Figure 4B), suggesting that FDPMP exerts favorable renoprotective effects against HFD-induced renal injury.

In the heart, myocardial disorganization, cardiomyocyte swelling, lipid accumulation, and inflammatory infiltration were observed in the model group, indicating that HFD could induce myocardial tissue injury and inflammatory responses. These changes were substantially reduced following FDPMP and simvastatin treatment, with myocardial fibers arranged in a more orderly manner, lipid deposition diminished, and inflammatory infiltration markedly resolved (Figure 4C), further confirming the cardioprotective effects of FDPMP.

Collectively, these findings demonstrate that FDPMP effectively attenuates multi-organ injury in the liver, kidney, and heart induced by HFD, and its tissue-protective effects are likely closely associated with its broad-spectrum anti-inflammatory and antioxidant properties, which are consistent with the reported bioactivities of FDPMP in previous studies [22,32]. However, the specific molecular mechanisms underlying the protective effects of FDPMP in the kidney and heart warrant further investigation, which represents an important direction for future in-depth exploration.

### 3.5. FDPMP Regulates Cholesterol Metabolism-Related Proteins in Hyperlipidemic Mice

Metabolomic analysis suggested significant alterations in lipid metabolism, particularly cholesterol-related pathways, in HFD-fed mice.

**Figure 5:**
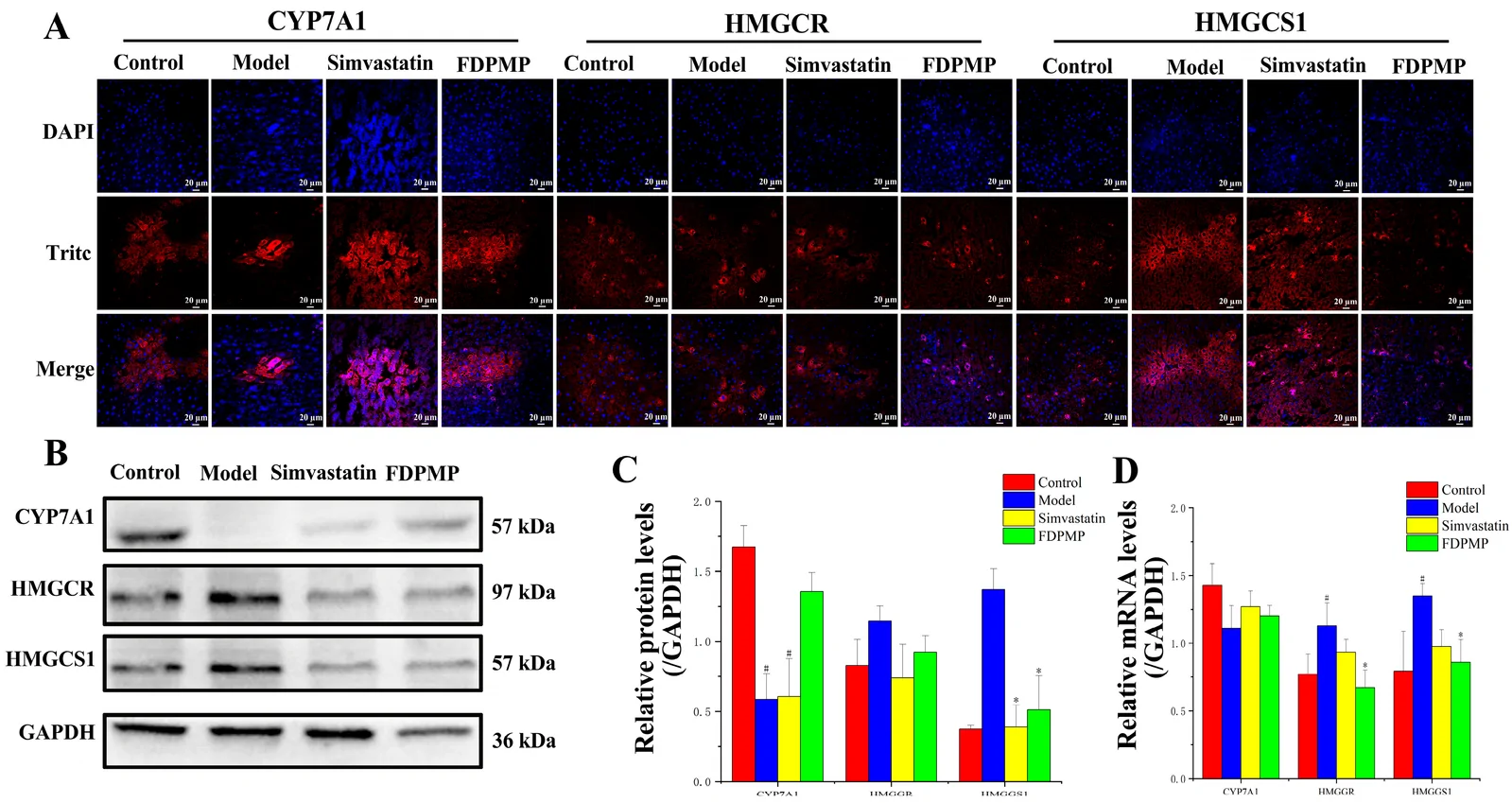
Effects of FDPMP on cholesterol metabolism-related proteins and gene expression in liver tissues (*n* = 6). (**A**) Immunofluorescence staining of CYP7A1, HMGCR, and HMGCS1(20x); (**B**,**C**) Protein expression levels determined by Western blot analysis; (**D**) Relative mRNA expression levels measured by RT-qPCR. Note: ^#^ *p* < 0.05, vs. control group; * *p* < 0.05, vs. model group. The original, uncropped western blot images are provided in Supplementary File S1. The original, uncropped Western blot images from a separate verification experiment are provided in Supplementary File S2.

Immunofluorescence staining showed that CYP7A1 expression was markedly reduced in the model group and significantly increased following FDPMP and simvastatin treatment. In contrast, the expression levels of HMGCR and HMGCS1 were elevated in the model group and decreased after treatment (Figure 5A). Western blot analysis confirmed these findings at the protein level, demonstrating significant modulation of CYP7A1, HMGCR, and HMGCS1 expression following FDPMP and simvastatin treatment (Figure 5B,C). Consistently, RT-qPCR results showed that CYP7A1 mRNA expression was significantly upregulated in treatment groups, while HMGCR and HMGCS1 expression levels were significantly reduced and approached those of the control group (Figure 5D).

These results indicate that FDPMP regulates key enzymes involved in cholesterol synthesis and bile acid metabolism. The observed modulation of cholesterol metabolism-related genes is consistent with the known bioactivities of *P. manillensis* components. Previous studies have demonstrated that the protein extract of *P. manillensis* lyophilized powder exhibits significant lipid-lowering effects by reducing serum TC, TG, and LDL-C levels while increasing HDL-C in ApoE^−/−^ mice [32]. Furthermore, recent evidence has shown that *P. manillensis* can lower blood lipid levels and exert anti-thrombotic properties through modulation of the PI3K/AKT and NF-κB signaling pathways [33]. Given that these signaling pathways are closely associated with the transcriptional regulation of HMGCR, HMGCS1, and CYP7A1, it is plausible that the bioactive peptides and proteins in FDPMP contribute to the coordinated regulation of cholesterol synthesis and bile acid metabolism observed in this study. These findings collectively suggest that the regulatory effects of FDPMP on cholesterol homeostasis are mediated, at least in part, by its active proteinaceous.

### 3.6. Effects of FDPMP on Foam Cell Formation and Lipid Metabolism in RAW264.7 Macrophages

**Figure 6:**
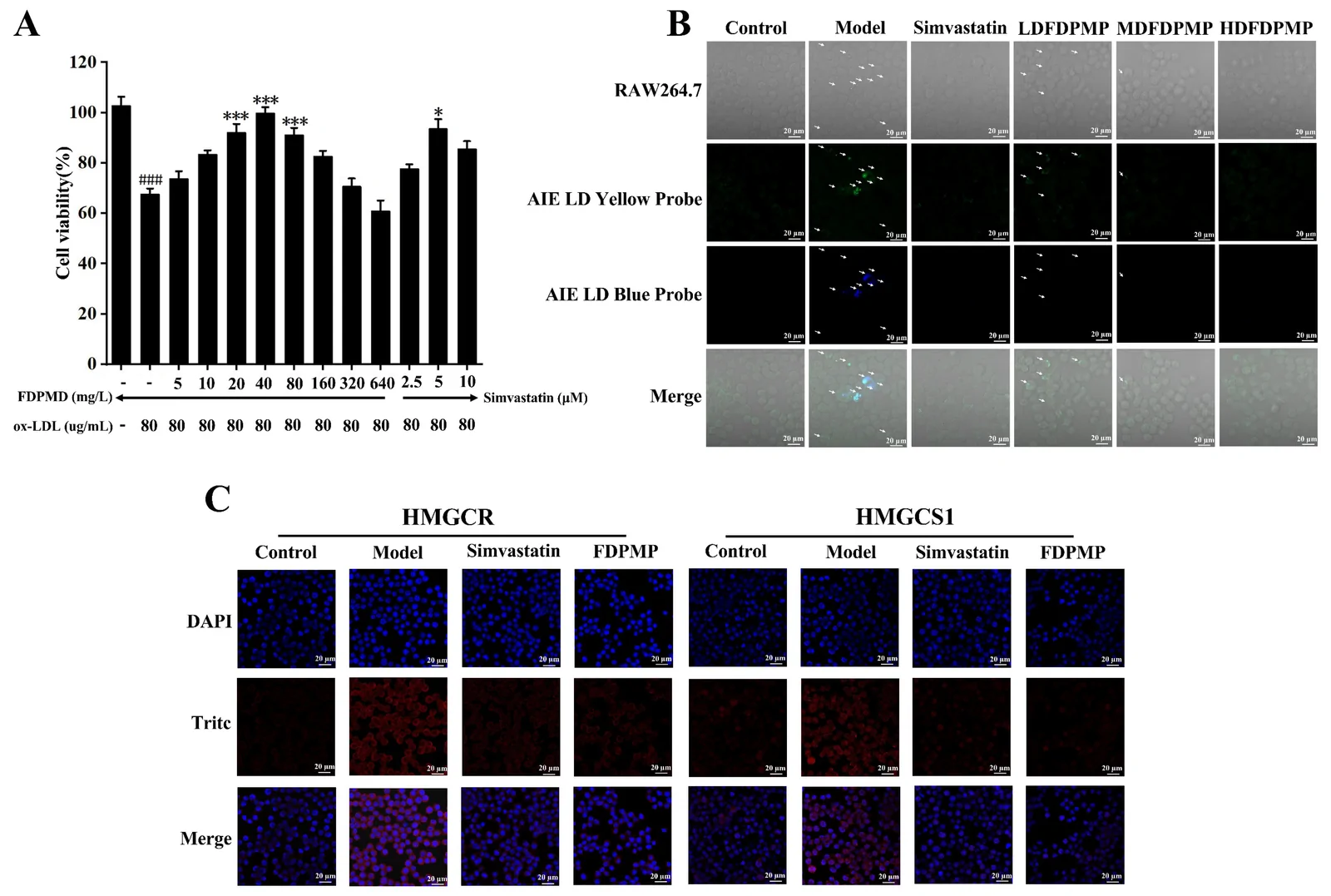
Effects of FDPMP on cell viability, lipid accumulation, and foam cell formation in RAW264.7 macrophages (*n* = 6). (**A**) Cell viability assessed by CCK-8 assay. (**B**) Intracellular lipid accumulation and oxidative stress visualized by fluorescence staining. (**C**) Immunofluorescence analysis of HMGCR and HMGCS1 expression. Note: ^###^ *p* < 0.001 vs. control group; * *p* < 0.05, *** *p* < 0.001 vs. model group. White arrows indicate lipid droplet accumulation and oxidative stress.

Cell viability assays showed that ox-LDL (80 μg/mL) significantly reduced RAW264.7 cell viability, whereas FDPMP treatment (10–160 mg/L) significantly improved cell viability, with the highest effect observed at 40 mg/L (Figure 6A). A cell viability below 80% was considered indicative of cytotoxicity [34].

Fluorescence staining revealed minimal lipid accumulation in control cells, whereas ox-LDL treatment induced marked intracellular lipid droplet accumulation and oxidative stress. Both FDPMP and simvastatin treatments significantly reduced lipid accumulation and oxidative stress in a dose-dependent manner (Figure 6B).

I Immunofluorescence analysis further demonstrated that ox-LDL significantly increased the expression of HMGCR and HMGCS1, while FDPMP and simvastatin treatments reversed these changes (Figure 6C).

These findings indicate that FDPMP inhibits ox-LDL-induced foam cell formation and modulates lipid metabolism in macrophages. The observed protective effects are consistent with the known bioactivities of bioactive peptides and proteins derived from *P. manillensis*. Previous studies have identified that the protein extract of *P. manillensis* lyophilized powder can reduce serum uric acid levels and modulate metabolic pathways through multi-target mechanisms [35]. Furthermore, bioactive peptides from *P. manillensis* have been reported to possess antioxidant activity, as evidenced by their ability to reduce reactive oxygen species (ROS) levels [32]. In macrophages, active peptides from various sources have been shown to regulate immune responses through the NF-κB signaling pathway, thereby modulating inflammatory cytokine secretion and oxidative stress [33]. Given that foam cell formation is driven by oxidative stress and lipid overload, it is plausible that the antioxidant and anti-inflammatory peptides and proteins in FDPMP contribute to the suppression of lipid accumulation, reduction in oxidative stress, and downregulation of HMGCR and HMGCS1 expression observed in this study. These findings collectively suggest that the inhibitory effects of FDPMP on foam cell formation are mediated, at least in part, by its active proteinaceous components.

## 4. Discussion

In the present study, we provide integrated in vivo, in vitro, and metabolomic evidence demonstrating that FDPMP exerts a protective effect against HFD-induced HL [36]. Mechanistically, FDPMP treatment was associated with coordinated changes in the expression of key cholesterol metabolism-related genes and proteins, characterized by upregulation of the bile acid-generating enzyme CYP7A1 and downregulation of the biosynthetic enzymes HMGCR and HMGCS1 [37]. These molecular changes suggest a potential dual regulatory effect on cholesterol synthesis and excretion, although direct functional measurements, such as bile acid levels and cholesterol excretion, are required to establish causal relationships. This regulatory pattern was accompanied by attenuation of oxidative stress, inhibition of foam cell formation, and alleviation of multi-organ injury, collectively supporting FDPMP as a multi-target metabolic regulator with therapeutic potential for HL and related disorders.

The improvement of systemic lipid profiles observed in this study reflects a global restoration of lipid metabolic balance rather than isolated modulation of individual lipid parameters. FDPMP significantly reduced serum TG, TC, and LDL-C levels while increasing HDL-C in ApoE^−/−^ mice, indicating a comprehensive lipid-lowering effect. Importantly, histopathological analyses further revealed that FDPMP markedly alleviated lipid deposition and structural damage in the liver, kidney, and heart. Given that HL exerts systemic pathological effects extending beyond the vasculature [38,39], such multi-organ protection highlights the broader metabolic benefits of FDPMP. These findings are consistent with the concept that effective HL interventions should not only normalize circulating lipid levels but also mitigate organ-specific pathological consequences [40,41].

Oxidative stress is a central driver of HL progression and atherosclerosis, linking lipid overload to inflammation and cellular injury [42]. In agreement with previous studies [43,44,45,46], HFD-induced HL in this study was characterized by elevated lipid peroxidation and impaired antioxidant defense. FDPMP treatment significantly enhanced the activities of key antioxidant enzymes, including GSH-Px and SOD, while reducing MDA levels, indicating restoration of redox homeostasis. Given that oxidative stress promotes LDL oxidation and accelerates foam cell formation, the antioxidant effects of FDPMP likely contribute to its downstream protective roles in inhibiting atherogenic processes [47,48]. Thus, modulation of oxidative stress represents an important complementary mechanism underlying the lipid-lowering efficacy of FDPMP. Beyond its antioxidative effects, FDPMP also modulated the expression of key enzymes governing cholesterol metabolism, as discussed below.

A major finding of this study is that FDPMP treatment correlated with significant changes in the expression of key enzymes involved in cholesterol metabolism. At the molecular level, FDPMP downregulated HMGCR and HMGCS1, key rate-limiting enzymes in the mevalonate pathway responsible for endogenous cholesterol production [49,50,51]. Overactivation of this pathway under HFD conditions leads to excessive cholesterol accumulation and contributes to HL pathogenesis [52]. Notably, most current lipid-lowering strategies, particularly statins, primarily target cholesterol synthesis through HMGCR inhibition [53]. In contrast, FDPMP exerted coordinated regulatory effects on both synthesis and excretion pathways at the transcriptional and protein levels, representing a mechanistically distinct and potentially more comprehensive approach for restoring cholesterol homeostasis. In contrast, FDPMP exerted coordinated regulatory effects on both synthesis and excretion pathways at the transcriptional and protein levels, a regulatory profile that differs from the single-target action of statins.

Untargeted metabolomics further revealed the multidimensional regulatory effects of FDPMP on systemic metabolism. HFD feeding significantly altered metabolites associated with choline metabolism, glycerophospholipid metabolism, and arachidonic acid metabolism—pathways closely linked to membrane lipid remodeling, inflammatory signaling, and lipid transport [54]. Specifically, HFD induced marked upregulation of glycerophosphocholines and arachidonic acid metabolites, reflecting enhanced phospholipid turnover and pro-inflammatory lipid signaling. FDPMP treatment effectively reversed these changes, downregulating glycerophosphocholine and arachidonic acid metabolites while modulating choline-associated pathways. These shifts suggest that FDPMP may reduce phospholipid turnover and lipid transport, thereby limiting cholesterol accumulation and lipid deposition—a finding consistent with previous reports that modulation of choline metabolism attenuates atherosclerotic progression [13,55,56]. Together, the metabolomic data indicate that FDPMP not only regulated cholesterol metabolism-related pathways but also reshaped broader metabolic networks associated with HL.

The inhibition of ox-LDL-induced foam cell formation by FDPMP further highlights its potential anti-atherosclerotic effects. Foam cell formation, resulting from excessive uptake of modified lipoproteins by macrophages, is a hallmark of early atherosclerotic lesion development [57]. In this study, FDPMP significantly reduced intracellular lipid accumulation and oxidative stress in RAW264.7 macrophages which may contribute to the inhibition of foam cell formation. This effect is likely mediated by the combined regulation of cholesterol metabolism and redox balance, as intracellular cholesterol overload and reactive oxygen species synergistically promote foam cell development [13,58]. These in vitro findings complement the in vivo results and provide mechanistic support for the role of FDPMP in preventing early atherogenic events.

Compared with conventional lipid-lowering drugs, FDPMP exhibits distinct advantages in terms of its multi-target regulatory capacity. Statins, such as simvastatin, effectively reduce cholesterol levels primarily through inhibition of HMGCR [40], but their single-target mechanism may not fully address the complex metabolic disturbances induced by HFD. In contrast, FDPMP simultaneously modulates cholesterol synthesis, bile acid metabolism, oxidative stress, and multiple metabolic pathways, suggesting a broader therapeutic spectrum. This multi-pathway regulation is further supported by metabolomic evidence showing coordinated changes in lipid- and amino acid-related metabolic networks [59]. Such systems-level modulation may be particularly beneficial for complex metabolic disorders like HL, where multiple pathological processes are intertwined.

Histological analyses further confirmed that FDPMP markedly alleviated HFD-induced hepatic steatosis, renal tubular damage, and myocardial structural abnormalities, consistent with the multi-organ protection described earlier. These findings are consistent with previous studies on traditional medicinal compounds, such as *Salvia miltiorrhiza* and classical herbal formulations, which exert protective effects against metabolic organ damage [41,60]. The ability of FDPMP to simultaneously improve lipid metabolism, reduce oxidative stress, and protect multiple organs further underscores its potential as a comprehensive therapeutic agent for HL-associated complications.

Despite these promising findings, several limitations should be acknowledged. First, the current design employed WT+SD as controls and ApoE^−/−^+HFD as treatment groups, but lacked ApoE^−/−^+SD and WT+HFD groups, which confounds genotype, diet, and treatment effects. While the ApoE^−/−^+HFD model is a well-validated “gold standard,” a full factorial design would be required to definitively parse the contribution of each factor and their interactions. Second, we did not perform direct functional measurements—such as bile acids, fecal sterols, or hepatic cholesterol content—to confirm enhanced cholesterol excretion or reduced biosynthesis; thus, our conclusions are correlative and require further functional validation. Third, further validation in additional models and clinical settings is needed [59]. Fourth, the potential involvement of gut microbiota was not explored and warrants future investigation [61]; Fifth, as an untargeted metabolomics approach was used, metabolite identifications were based on spectral matching without authentic standards (MSI Level 2), and three unannotated features were excluded from pathway interpretation. Targeted metabolomics using authentic standards—particularly for choline metabolites, glycerophospholipids, arachidonic acid products, and bile acids—is warranted to quantitatively confirm these findings. Addressing these limitations will help to further clarify the therapeutic mechanisms and translational potential of FDPMP.

## 5. Conclusions

In conclusion, this study demonstrates that FDPMP exerts potent lipid-lowering and metabolic protective effects through coordinated regulation of cholesterol synthesis and excretion, coupled with modulation of oxidative stress and systemic metabolic pathways. By integrating multi-level evidence, our findings not only elucidate the mechanistic basis underlying the traditional medicinal use of *P. manillensis* but also highlight FDPMP as a promising natural therapeutic candidate for HL and related metabolic disorders.

## Figures and Tables

**Table 1:** RT-qPCR primer sequences.

Gene	Species	Primers
GAPDH	Mouse	F: GAAGGTCGGTGTGAACGGATTTG
		R: CATGTAGACCATGTAGTTGAGGTCA
CYP7A1	Mouse	F: TTGCTACTTCTGCGAAGGCA
		R: TCCGTGAGGGAATTCAAGGC
HMGCR	Mouse	F: GTCTGAACTGGAACATGGGC
		R: TTCATCCTCCACAAGACAATGC
HMGCS1	Mouse	F: AAGCTCAGAGAGGACACCCAT
		R: CGAGCGTAAGTTCTTCTGTGC

**Table 2:** Differentially expressed endogenous metabolites identified by UHPLC–QTOF/MS among the control, model, and FDPMP groups.

No.	Rt (min)	*m*/*z*	Metabolites	Control vs. Modle			Modle vs. PMFDP		
				VIP	FC	Trend	VIP	FC	Trend
1	0.63	82.10	-	1.45	0.39	↓ ^##^	1.65	2.01	↑ **
2	9.30	82.50	-	1.33	0.59	↓ ^#^	1.72	6.51	↑ *
3	8.29	76.3	Lipids and lipid-like molecules	1.45	4.47	↑ ^##^	1.45	0.32	↓ **
4	7.72	62.80	Amino acids, peptides, and analogues	1.45	3.55	↑ ^#^	1.69	0.31	↓ *
5	0.98	70.30	Amino acids, peptides, and analogues	1.39	0.47	↓ ^#^	1.63	1.71	↓ *
6	1.13	94.70	Fatty acid esters	1.40	0.37	↓ ^##^	1.69	1.91	↑ *
7	4.24	71.90	Alloxazines and isoalloxazines	1.27	1.52	↑ ^#^	1.73	0.64	↓ *
8	7.505	82.1	Glycerophosphocholines	1.47	2.53	↑ ^##^	1.88	0.65	↓ ***
9	9.31	51.6	Amino acids, peptides, and analogues	1.30	0.77	↓ ^#^	1.58	0.79	↓ *
10	7.16	72.70	Glycerophosphocholines	1.25	0.75	↓ ^#^	1.81	0.58	↓ **
11	7.38	82.80	Glycerophosphocholines	1.43	1.82	↑ ^#^	1.84	0.67	↓ ***
12	3.94	88.4	Amino acids, peptides, and analogues	1.41	0.22	↓ ^##^	1.79	3.40	↑ **
13	8.399	53.6	Vitamin D and derivatives	1.46	6.91	↑ ^##^	1.65	0.48	↓ *
14	0.677	60.7	Sulfones	1.49	0.19	↑ ^###^	1.64	1.56	↓ *
15	8.81	58.1	Glycerophosphocholines	1.39	0.81	↑ ^#^	1.83	46.59	↓ *
16	2.139	89.00	Amino acids, peptides, and analogues	1.44	2.33	↓ ^##^	1.60	2.33	↑ *
17	1.61	69.50	Amino acids, peptides, and analogues	1.39	0.50	↓ ^##^	1.76	1.51	↑ *
18	0.63	55.20	-	1.41	0.63	↓ ^##^	1.78	1.33	↑ **
19	0.60	63.30	Carbohydrates and carbohydrate conjugates	1.43	1.33	↓ ^##^	1.83	37.96	↑ *
20	1.71	89.20	Amino acids, peptides, and analogues	1.40	0.45	↓ ^#^	1.69	2.73	↑ *
21	3.59	77.30	Fatty acid esters	1.38	0.47	↓ ^#^	1.71	2.22	↑ *

Note: ^#^ *p* < 0.05, ^##^ *p* < 0.01, ^###^ *p* < 0.001 vs. control group; * *p* < 0.05, ** *p* < 0.01, *** *p* < 0.001 vs. model group. ↑ indicates increased abundance; ↓ indicates decreased abundance.

## Data Availability

All non-targeted metabolomic data used in this publication have been deposited to the EMBL-EBI MetaboLights database with the identifier MTBLS11268.

## Supplementary Materials

The following supporting information can be downloaded at: https://www.mdpi.com/article/10.3390/biom16081168/s1, File S1: Cropped Western blot bands as presented in Figure 5B of the main text; File S2: Original uncropped Western blot membrane from a separate experiment using the same samples and antibody (for verification purposes).
